# Effects of Pre-Workout Multi-Ingredient Supplement on Anaerobic Performance: Randomized Double-Blind Crossover Study

**DOI:** 10.3390/ijerph17218262

**Published:** 2020-11-09

**Authors:** Piotr Kaczka, Amit Batra, Katarzyna Kubicka, Marcin Maciejczyk, Agata Rzeszutko-Bełzowska, Iwona Pezdan-Śliż, Monika Michałowska-Sawczyn, Marta Przydział, Artur Płonka, Paweł Cięszczyk, Kinga Humińska-Lisowska, Tomasz Zając

**Affiliations:** 1 Department of Sport Nutrition, Academy of Physical Education in Katowice, ul. Mikołowska 72a, 40-065 Katowice, Poland; amit@op.pl (A.B.); kz.kubicka@gmail.com (K.K.); pbcz@awf.katowice.pl (T.Z.); 2 Department of Physiology and Biochemistry, University of Physical Education in Krakow, al. Jana Pawła II 78, 31-571 Kraków, Poland; marcin.maciejczyk@awf.krakow.pl; 3 Faculty of Physical Education, University of Rzeszow, ul. Towarnickiego 3, 35-010 Rzeszów, Poland; arzeszutko@ur.edu.pl (A.R.-B.); ipezdan@ur.edu.pl (I.P.-Ś.); mprzydzial@ur.edu.pl (M.P.); plonka77@op.pl (A.P.); 4 Department of Molecular Biology, Gdansk University of Physical Education and Sport, ul. Kazimierza Górskiego, 80-336 Gdańsk, Poland; monikamichalowska@op.pl (M.M.-S.); cieszczyk@poczta.onet.pl (P.C.); kinga.huminska-lisowska@awf.gda.pl (K.H.-L.)

**Keywords:** pre-workout supplementation, resistance training, caffeine, multi-ingredient performance supplement, MIPS, anaerobic performance

## Abstract

Background: The purpose of this research was to investigate the acute effects of a pre-workout supplement on anaerobic performance in resistance-trained men. Methods: Twenty-three men underwent three randomized, double-blind testing sessions separated by a seven-day break. The participants performed three tests: isokinetic strength, three repetition maximum (3-RM) strength and Wingate. Statistical analysis was conducted in R environment. Linear mixed models were estimated via R package lme4. Results: Mean T@0.2 s was significantly greater in supplemented condition for right and left knee flexors (PL: 103.2 ± 37.6 Nm; supplemented condition: 131.8 ± 29.3 Nm (*p* = 0.001)), and PL: 103.7 ± 39.3; supplemented condition: 129.4 ± 28.4 (*p* = 0.001)). T@0.2 s for right and left knee extensors (PL: 202.6 ± 58.6 Nm; supplemented condition: 237.2 ± 54.7 Nm (*p* = 0.001); PL: 203.3 ± 63.2 Nm, supplemented condition: 229.8 ± 50.8 Nm (*p* = 0.002)). Significant difference was in mean anaerobic power between supplemented and PL condition for right and left knee flexors (*p* = 0.002, *p* = 0.005) and for right and left knee extensors (*p* = 0.001 and *p* = 0.002). TTP was significantly shorter in supplemented condition for both sides knee flexors (*p* = 0.002). There was a significant difference for mean power in the Wingate test (placebo: 8.5 ± 0.6 W/kg; supplemented condition: 8.7 ± 0.5 W/kg (*p* = 0.038)). Mean 3-RM was significantly greater in supplemented condition (*p* = 0.001). Conclusions: The supplement significantly improves upper and lower body strength and power output in resistance-trained men.

## 1. Introduction

The physiological effect of a training session is dependent upon the quality of the work undertaken (general information); hence, athletes constantly search for methods to enhance the training outcome. Consequently, pre-workout formulations are becoming an increasingly popular class of dietary supplements among athletes. The prevalence of supplementation among athletes has been estimated at 37% to 89% [1], where the energy drinks were the most popular supplements next to multi-vitamins in the young adult population (18–35 years) [2]. However, pre-workout supplements take many forms and are based on multiple active ingredients and blends and in the majority of cases, the efficacy and safety has not been established [3,4].

Pre-training supplements are multi-ingredient compositions (MIPS) aimed usually at enhancing strength, shortening reaction time and eliciting focus [5]. For example, it is believed that substances such as caffeine, beta-alanine, L-citrulline, L-arginine, L-tyrosine, taurine and herb and botanical ingredients such as guarana extract (containing caffeine), barley extract (containing hordenine), cayenne pepper extract (containing capsaicin), black pepper extract (containing piperine) and Huperzia serrata extract, which target different physiological mechanisms, may elicit synergistic effect and in turn improve athletic performance [5]. A synergistic effect occurs when two or more substances combined with each other create an effect greater than each single of them could have exhibited by itself.

The most common ingredient of MIPS is caffeine, which has been shown to be an effective ergogenic aid for endurance exercise by delaying fatigue and increasing time to exhaustion [6,7]. However, caffeine’s effect on anaerobic performance (strength-power) is more equivocal, with some studies indicating benefits [8,9], while others do not demonstrate any significant change in resistance exercise performance [10]. There is a lack of significant findings for caffeine ingestion and lower body strength as compared to upper body performance [11]. Caffeine is often combined with taurine in several products known as energy drinks. Baum et al. [12] reported that one of them, which contains taurine and caffeine, as compared to a similar drink without taurine, favorably influences cardiac parameters, mainly an increased stroke volume, during recovery after exercise. It is believed that taurine can enhance muscle excitation-contraction coupling by maintaining intracellular calcium homeostasis [11]. Bakker et al. [13] used mechanically skinned fast-twitch fibers and showed greater force production during taurine in vitro treatment. Previous experiments on humans have shown that taurine ingestion alone did not improve cycling time–trial performance, despite a 16% increase in total body fat oxidation [14].

High energy production within the muscle tissue and high ATP turnover results in the accumulation of H+ ions. Lowering pH value is associated with slower energy production due to compromised oxidative phosphorylation and phosphocreatine resynthesis. It is stated that increasing the buffering ability of the endogenous systems is effective in increasing systemic control of the pH value and maintaining physical exercise. Beta-alanine has been shown to significantly elevate carnosine levels in both type I and type II human muscle fibers and act as an intracellular buffer [13]. Regular use of beta-alanine has been reported to improve buffering capacity of skeletal muscle and enhance power output during high-intensity exercise due to increasing levels of muscle carnosine [14,15]. Therefore, substances with the potential ability of increasing extracellular buffering capacity are constantly being sought [15,16]. Additionally, the recommended dose of beta-alanine loading is 2–5 g and a minimum 2–4 weeks of supplementation is needed to increase muscle carnosine levels [17].

Tyrosine supplementation is assumed to maintain optimum levels of brain neurotransmitters contributing to the optimal performance through higher motivation levels together with decreased fatigue and associated with lower ratings of perceived exertion [18]. Amino acids, L-arginine and L-citrulline found in the supplement formula are believed to be potent precursors of NO (nitric oxide), which plays a crucial role in blood flow, muscle energy metabolism and mitochondrial oxidation during exercise [19,20]. On the other hand, oral intake of L-citrulline increases not only L-citrulline, but also plasma L-arginine levels, and thus, is considered to be more effective for enhancing sport performance [21,22]. Acute intake of L-citrulline malate was reported to increase the number of repetitions to exhaustion during resistance exercise and decrease muscle soreness in 24 h and 48 h after high volume resistance-training.

Huperzia serrata extract works mainly by inhibiting the enzyme acetylcholinesterase, which breaks down acetylcholine [23,24]. Huperzine was reported to significantly increase the amplitude of muscle contraction induced by nerve stimulation [25]. Thus, one could suggest that huperzine may improve neuromuscular strength potential, alertness and focus by increasing the endplate potential and brain neurotransmitters levels [26].

Capsaicin and piperine are natural pungent-tasting compounds found in chili and black pepper, respectively. Those ingredients are found to be TRP1 agonists, which stimulate the sympathetic nervous system (SNS) and increase the energetic metabolism in humans through sensory nerve stimulation [27]. Moreover, TRPA1 agonists have been shown to induce adrenaline secretion. Thus, it can be hypothesized that these compounds may act synergistically with caffeine [28]. Moreover, pepper-derived alkaloids such as capsaicin and piperine are found to have thermogenic and energy-providing effects, which are triggered by activation of thermoreceptors and release of catecholamines [29]. Finally, barley-derived hordenine, which is also found in citrus aurantium, may have an influence on adrenergic receptors by stimulating the release of noradrenaline [28].

Based on the physiological properties of the individual substances listed above, recently, a new MIPS has been developed with a view to achieve synergistic action of the active substances included in the formulation. The supplement contains ingredients that are purported to stimulate the central nervous system and augment strength and power performance. We hypothesized that the tested supplement can significantly affect the anaerobic physical performance. We also expected that the active ingredients (citrulline, taurine, beta alanine, L-arginine, L-tyrosine and plants extracts of hordenine, Huperzia serrata, black and cayenne pepper) could impact significant effect. If this was the case, it would be characterized inter alia by greater strength and shorter time to peak torque (TTP) compared to placebo treatment.

It should be noted that commercially available pre-workout supplements with a number of various ingredients do not have estimated effectiveness for the finished formulation concerning both active and additional substances. Therefore, the purpose of this investigation is to examine the acute effects of the commercially available pre-workout supplement on anaerobic performance in resistance-trained men. It should be emphasized that estimating the influence of MIPS on maximal strength was not the main purpose and primary goal in many of the previous studies [5,30].

## 2. Materials and Methods

### 2.1. Materials

Twenty-three resistance-trained men (27 ± 7.4 years; 88 ± 10.7 kg; 179 ± 6 cm) with three years of resistance training experience were qualified for the study. All the subject had similar training experience focused on anaerobic performance with strength training three times a week, ∼100 min per training session. During the course of the study the participants underwent three testing sessions administered in randomized and double-blind fashion. The subjects were asked to follow a similar training scheme for eight weeks prior to the beginning of the study. The main part of each training consisted of 4 × 3–5 repetitions of a single exercise for each muscle group, with ∼80% of 1-RM, 3 min rest intervals. This volume was based on the participants’ training experience. It is also known that on average 80% of the 1RM applies to 2–5 repetition range [31]. Following an explanation of all procedures, risks and benefits associated with the study, each subject gave his written consent prior to participation. The study was approved by the Ethical Committee of the University School of Physical Education in Katowice (Katowice, Poland; Resolution No. 2/2018) and conformed to the ethical requirements of the 1975 Helsinki Declaration. Subjects were also required to be free of any nutritional supplements or ergogenic aids for the two weeks preceding the study, and were asked to refrain from taking any additional supplement during the duration of the study.

### 2.2. Methods

This was randomized, double-blind, crossover study. All subjects attended a familiarization session for all of the test exercises one week before testing. To reduce the effect of any caffeine tolerance, they were instructed not to consume caffeine containing products 24 h before testing. This time was estimated due to caffeine’s half-time and elimination rate [32,33,34]. Subjects were also asked to abstain from heavy exercise and alcohol consumption during the period of the experiment. Participants were randomly divided into two groups and received either complex formulation or placebo solution. In addition, subjects were instructed not to eat or drink for three hours prior to each trial. Subjects reported to the Performance Laboratory of Academy of Physical Education in Katowice on three separate days (Saturdays; familiarization session and two testing sessions) with seven days apart between the test days. Following a 10-min resting period in the seated position, subjects were randomly provided with either the flavored water placebo (PL—water and flavors only) or the supplement, which is commercially marketed as Knockout 2.0^®^ (Olimp Laboratories Sp. z o.o., Dębica, Poland). The supplement consisted of 9.6 g powder mixed with water (250 mL) containing: L-citrulline (3 g), beta-alanine (2 g), taurine (750 mg), L-arginine (500 mg), L-tyrosine (500 mg), anhydrous caffeine (300 mg), guarana extract (200 mg), barley–derived hordenine extract (150 mg), capsaicin extract (25 mg), black pepper extract (7.5 mg) and Huperzia serrata extract (3 mg). After consumption of either PL or the supplement solution, subjects took a 15 min rest prior to commencing the warm-up and exercise testing. The warm-up lasted for 20 min and was divided into two phases. The first phase was a 10-min general warm-up with light stationary cycling at a self-selected cadence. The second phase consisted of dynamic body-weight movements (eight minutes) and light stretching exercises (six stretching exercises performed in two series of 10 s each), with a total 2 min of static stretching for the main muscle groups involved in test exercises [33]. The last five minutes of the preparation were dedicated to proper Biodex chair height and attachments alignment. The time from the intake of the solution to the start of the test was based on caffeine’s half-time and elimination rate [32,33,34]. We decided to use this value because caffeine is the best studied among all the ingredients used and has the best known pharmacological effects. This alkaloid is not synthetized nor is naturally occurring in the body. After ingestion, it is rapidly and almost completely absorbed in humans, metabolized and excreted primarily as the well studied compound paraxanthine. Other substances used in the formulation, such as proteinogenic and non-proteinogenic aminoacids, are normally occurring in the human body. At the same time, plant ingredients used are standardized for one chosen compound but contain numerous other chemical substances, such as polyphenols that are not strictly quantified.

Hence, 40 min following the intake of the solution, subjects underwent testing procedures consisting of muscular isokinetic knee flexion/extension test, three repetition maximum upper body strength test—bench press (3-RM) and the Wingate anaerobic test (WAnT). The tests were always carried out in the mentioned order. Each performance assessment was separated by a five-minute rest period. On the subjects’ second and third visit to the laboratory, they were provided with the opposite treatment. The authors of the study decided to select such tests, because each test allowed the measurement of different parameters and complex examination: bench press—maximal strength, isokinetic test for knee joint flexors and extensors—peak torque, Wingate test—peak power, time to peak power, total work and lactate blood concentration. Different combination of the mentioned exercises would cause a greater overlap of local fatigue, and therefore, inconsistent results.

### 2.3. Isokinetic Test

The test was performed according strictly to Biodex protocol for isokinetic operating mode for the knee joint. Athletes were placed on the isokinetic dynamometer (Biodex Multi-joint System 4 PRO, Biodex Medical Systems Inc, Shirley, NY, USA) in a sitting position with hip flexion at 85° and the equipment axis aligned with the lateral condyle of the femur. Both arms were placed along the sides of the body, the trunk was stabilized against the backrest using chair belts, the thigh of the tested limb was fixed against the seat by means of a belt, and the contralateral limb was allowed to hang free. The tested leg was weighted to correct for the effects of gravity on the torque measured, according to the specifications of the Biodex Manual. To assess muscular performance, each participant was asked to perform alternating concentric contractions of the knee flexors and extensors within a range of motion of 85° (90° to 5° of flexion). During the test, every participant was instructed to exert maximum force throughout the entire range of motion. In addition, they were encouraged to go as fast as possible until the end of the assessment. Participants were allowed to familiarize themselves with the procedures before actual testing by performing three repetitions of the tested motion. Then, they performed a set of five repetitions at 60°/s. Variables collected during the test were: time to peak torque (TTP), described as measure of time from the start of muscular contraction to the point of the highest torque development (peak torque), peak torque (PT), highest muscular force output at any moment during a repetition, torque at 0.2 s (T@0.2 s), force developed in first 0.2 s from the start of contraction, and total work performed (Twork), the amount of work accomplished for the entire set of repetitions. When the coefficient of variation (CV) of the peak torque was higher than 10%, the athlete was allowed to recover and the set was repeated [35].

### 2.4. Maximal Strength Test

Subjects performed a three–repetition maximum (3-RM) test in the bench press exercise five minutes after completing the isokinetic strength test. Initially, they warmed-up by completing 12–15 repetitions on the standard barbell without any additional load (TechnoGym Bar, Cesena, Italy) followed by 12–15 repetitions with 40–60 kg load (according to each participant’s ability), at a self-selected cadence.

3 RM determination was carried out according to Baechle and Earle methods [36]. Two minutes of recovery was allotted between sets and 3_RM was performed for 3–6 sets. The test was performed on a flat bench with an integral bar rack. The start was a supine, five-point body contact position with the bar rack over the face. The grip was a little wider than the shoulder-width, closed and pronated. After moving the bar from the supports it was placed over the chest with elbows fully extended. Subsequently, the bar was lowered to touch the chest, forearms perpendicular to the floor and parallel to each other. No bouncing of the bar on the chest was permitted for the bench press exercise, as this would have artificially augmented strength results. The upward movement was performed in five-point body contact position without arching the back or raising the chest. The bar was pulled upward until the elbows were fully extended. After the last repetition the bar was racked. During the whole test a spotter was present.

### 2.5. Wingate Test

Wingate test procedure began with five–minute warm–up at 60–70 RPM cadence on Cyclus2 ergometer (BM elektronik-automation GmbH, Leipzig, Germany). After five minutes of recovery, each participant performed a 30 s supramaximal effort at an individually determined workload of 7.5% body mass. Subjects were instructed to accelerate as fast as possible to the highest attainable pedaling rate and to maintain the pace throughout the whole test duration, while remaining in a seated position. During the test, the following mechanical variables were collected: peak power (PP), mean power (MP), fatigue index (FI) and total work performed (Twork). The peak power achieved was defined as the highest power output achieved during the 30 s test, while mean power was defined as the average power achieved throughout the trial [37]. Time to peak power corresponds to the time needed to reach peak power from the beginning of the test. The fatigue index reflects the percent power decline during the trial [37]. The work performed was calculated basing on the total number of revolutions and force computed by Cyclus2 software. In the third minute of recovery, finger capillary blood (2 μL) was collected for plasma lactate measurement (Lactate Scout, EKF-Diagnostic GmbH, Leipzig, Germany).

### 2.6. Statistical Analysis

Analysis was conducted in R environment (version 3.3.2; The R Foundation for Statistical Computing, Topeka, KS, USA). Linear mixed models were estimated via R package *lme4*. Normality of data was ascertained via graphical methods (quantile-quantile plots). Levene’s test (based on median) showed that for all variables, variances were homoscedastic. Thus, data could be analyzed using parametric methods. In order to assess the significance of differences between conditions (MIPS vs. PL) the analysis of variance (ANOVA) with repeated measurements was used. Firstly, likelihood ratio tests with Benjamini-Hochberg FDR correction were used to screen out non-significant models. Afterwards, pairwise differences between MIPS and PL conditions were examined via Tukey’s HSD procedure (post-hoc analysis).

Effect size was estimated using marginal and conditional (pseudo-R^2^) linear association between standardized variables. Linear mixed models (with random intercepts and slopes) were applied. Firstly, likelihood ratio tests (with Benjamini Hochberg correction) were applied to the simple models (no condition effects) to assess the significance of the regression coefficient and effect size was estimated by marginal and conditional (pseudo-R^2^). Then, likelihood ratio tests (with Benjamini-Hochberg correction) were applied to compare simple and extended models (MIPS and PL conditions effects and interaction effect with the continuous predictor were added) to determine whether regression coefficient differ significantly between MIPS and PL conditions. Statistical significance was set at *p* < 0.05. All data are reported as mean ± standard deviations (SD).

## 3. Results

No subjects reported any adverse events or side-effects following ingestion of the supplement or placebo.

### 3.1. Isokinetic Test

**Figure 1:**
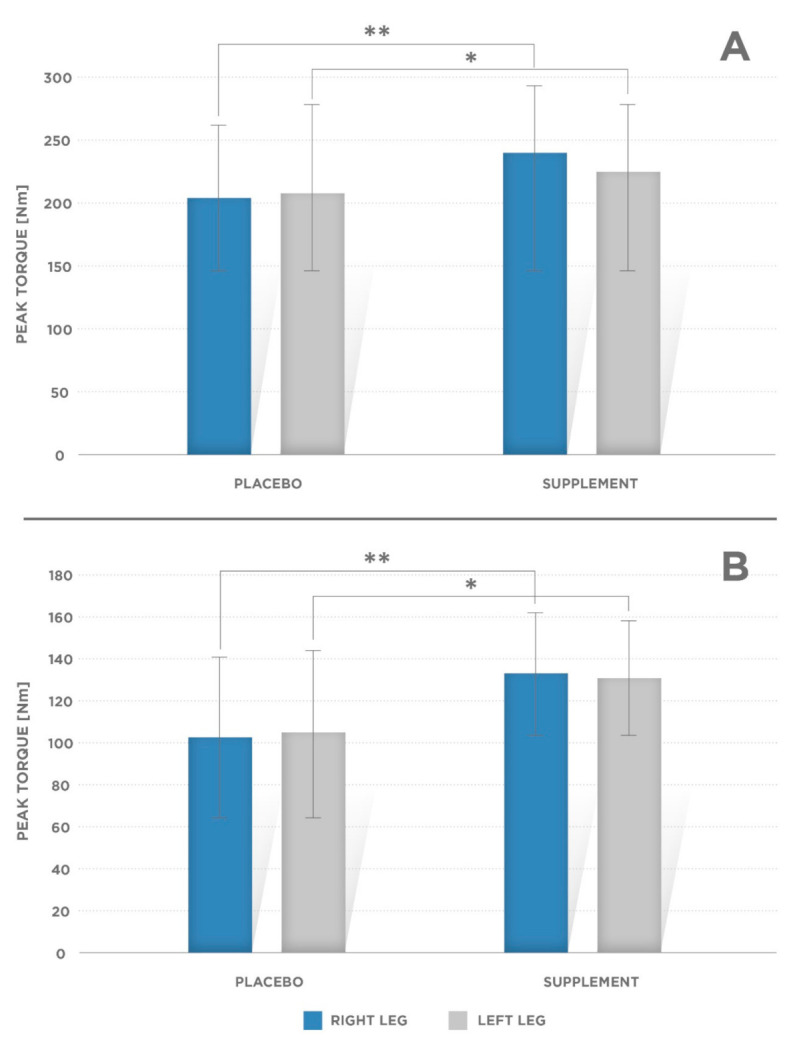
Mean values of torque (T@0.2s) at 60°/s at 0.2 s for (**A**) the right and left knee extensors. Significant difference was observed for the right leg (** *p* = 0.001) and left leg (* *p* = 0.002) and (**B**) for the right and left knee flexors. Significant difference was observed for the right leg (** *p* = 0.001) and left leg (* *p* = 0.002). Error bars indicate standard deviation (SD).

The mean values of knee peak torque (PT) developed by the knee extensors and flexors muscle groups (left and right side) were significantly greater in supplement (*p* = 0.001 for right and left leg flexors as well as for right leg extensors, and *p* = 0.002 for left leg extensors) compared to PL treatment (Figure 1A,B).

**Figure 2:**
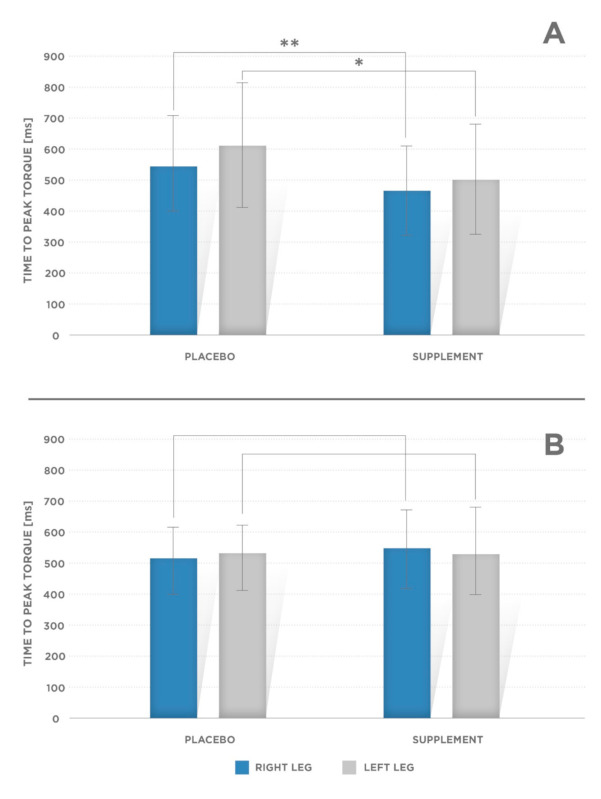
Mean values of time to peak torque (TTP) at 60°/s for (**A**) the right and left knee flexors. Significant difference was observed for the right leg (** *p* = 0.002) and left leg (* *p* = 0.002) and (**B**) for the right and left knee extensors. Significant difference for extensors was not observed for the right (*p* = 0.818) and left leg (*p* = 0.422). Error bars indicate standard deviation (SD).

**Figure 3:**
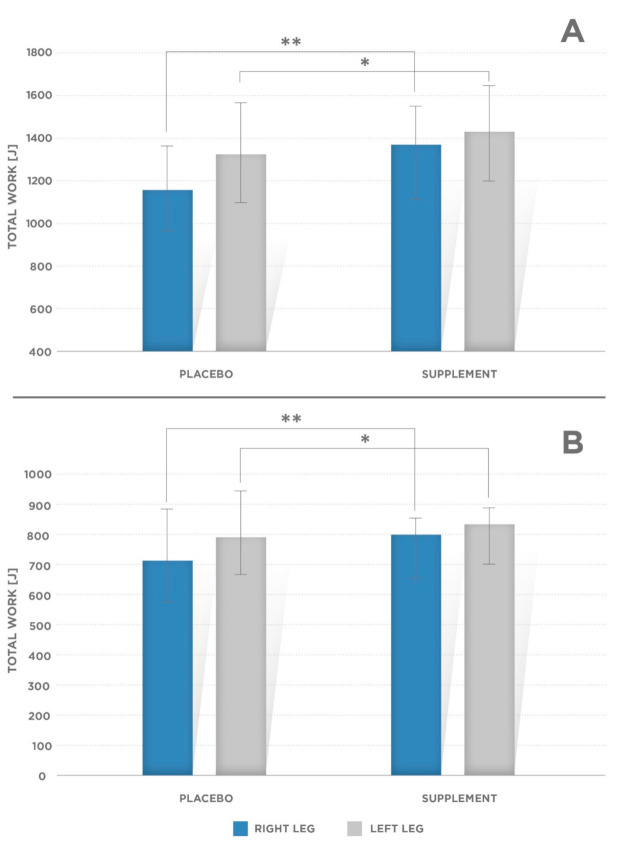
Mean values of total work (Twork) at 60°/s for (**A**) the right and left knee extensors. Significant difference was observed for the right leg (** *p* = 0.001) and left leg (* *p* = 0.002), and (**B**) for the right and left knee flexors. Significant difference was observed for the right leg (** *p* = 0.001) and left leg (* *p* = 0.002). Error bars indicate standard deviation (SD).

- Mean torque at 0.2 s (T@0.2 s) value for knee extensors were greater in supplemented than in placebo condition (Figure 1A, Table 1):
- Right side: PL: 202.6 ± 58.6 Nm; supplement condition: 237.2 ± 54.75 Nm (*p* = 0.001);
- Left side: PL: 203.3 ± 63.2 Nm, supplement condition: 229.8 ± 50.8 Nm (*p* = 0.002).
  Mean PT was significantly greater in supplement condition for knee flexors (Figure 1B):
- Right side: PL: 103.2 ± 37.6 Nm; supplement condition: 131.8 ± 29.3 Nm (*p* = 0.001);
- Left side: PL: 103.7 ± 39.3; supplement condition: 129.4 ± 28.4 (*p* = 0.001).
  Other mechanical variables obtained via isokinetic strength tests for the knee joint:
- Time to peak torque—TTP [ms] (Figure 2A, Table 1) for the knee flexors (*p* = 0.002 for the right and left leg);
- Total work—T_work_ [J] (Figure 3A,B, Table 1)—done for the knee extensors and flexors muscle conditions (left and right extremities; *p* = 0.002 and *p* = 0.005 for the right and left leg flexors, respectively, and *p* = 0.001 and *p* = 0.002 for the right and left leg extensors, respectively).

### 3.2. Maximal Strength Test

**Figure 4:**
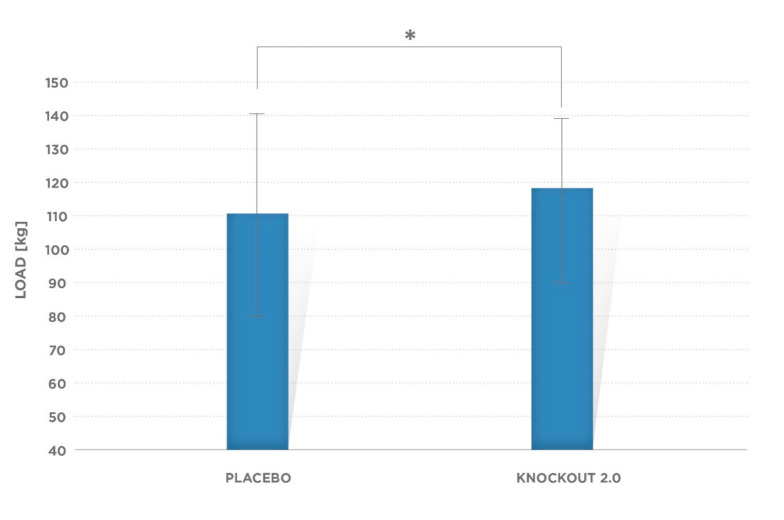
Mean values of 3-RM strength. There were observed significant difference (* *p* = 0.001) between supplement and placebo conditions. Error bars indicate standard deviation (SD).

Mean 3-RM strength for placebo treatment was 110.6 ± 29.75 kg, while for the supplement, subjects’ performance was 118.82 ± 29.89 kg, which demonstrated a statistically significant difference (*p* = 0.001; Figure 4).

### 3.3. Wingate Test 

The Wingate anaerobic test results are depicted in Table 2. Significant difference in mean power (MP) between supplement and PL treatment was observed (*p* = 0.038; diff: 0.18; 95 % CI: 0.02 to 0.34). No statistical difference was noticed between other variables presented in Table 2.

## 4. Discussion

The aim of this study was to investigate the acute effects of commercially available pre-workout supplement on anaerobic exercise in resistance-trained men. We hypothesized that the supplement could significantly and beneficially affect the anaerobic physical performance. We also expected that the active ingredients (citrulline, taurine, beta alanine, L-arginine, L-tyrosine and plants extracts of barley—Hordeum vulgare L., Huperzia serrata P.E., black pepper—Piper nigrum and cayenne pepper—Capsicum annuum) could impart significant effect, for example greater strength and shorter time to peak torque (TTP) comparing to placebo treatment.

The results of this study indicate that the ingestion of multi-ingredient pre-workout dietary supplement prior to physical exercise was effective in improving resistance and high–intensity performance. The results show that the supplement could delay fatigue and improve strength. The mean peak torque of muscle extensors and flexors increased significantly during isokinetic strength test with supplement ingestion. These results are consistent with previous findings in which isokinetic strength performance was improved due to caffeine ingestion [36,37]. It was also found that supplement significantly increased torque (extension and flexion) at 0–200 ms time interval, which is described as an improvement in the rate of force development (13% and 20% improvement for extensors and flexors, respectively) and has important implications for performance in sports where forces have to be applied rapidly [38,39,40]. Similar findings were reported in the study by Behrens et al. [41], who confirmed the supraspinal excitatory effect of caffeine on motor unit recruitment and rate coding. These results indicate that pre-training supplements based on caffeine may be helpful in ballistic–related exercises [40,41,42].

The ergogenic effects of caffeine during resistance exercise or high intensity exercise protocol have been seen in doses ranging from of 3–6 mg·kg^−1^ [11]. The average dosage of caffeine provided in this study was 3.4 mg·kg^−1^. In other studies, where 1-RM bench press strength exercise was improved, the amount of caffeine in caffeine-containing supplement administrated was slightly lower (201 mg per dose = 2.4 mg·kg^−1^) [31]. However, the improvement in the study was around 2.1%, which is clearly lower than the strength improvement seen in the current study (7%), which could indicate a better chosen composition of additional active ingredients, which caused a better ergogenic synergistic effect of the of the MIPS investigated in this study. Alternatively, this discrepancy in caffeine content—a dosage of 1 mg·kg^−1^—could be responsible for this strength improvement effect that was more than three times higher. Lastly, both causes could have played a role. A synergistic effect should be understood as a situation when two or more substances combined with each other create an effect greater than each single substance could have exhibited by itself.

In contrast, Astorino et al. [10] supplemented 6 mg·kg^−1^ of caffeine to resistance-trained men and did not observed any difference in 1-RM bench press performance. Interestingly, Williams et al. [42] combined caffeine with ephedrine before 1-RM bench press protocol and also did not observe any significant changes in performance. Nevertheless, the improvement of the resistance exercise performance due to caffeine or MIPS ingestion is documented by some but not all of the previous research. Regarding the levels of caffeine habituation, different testing protocols and caffeine dosages are potential contributory factors that may be responsible for different outcomes found in the scientific literature.

During the Wingate test we observed (Table 2) that only the mean anaerobic power was significantly improved (*p* < 0.05). No statistical differences in the other variables in Wingate test were observed. Nevertheless, we can suggest that the greater value of mean anaerobic power performance compared to PL was possibly due to enhanced anaerobic glycolysis in supplement trial [40,41]. It is possible that the onset of local and peripheral fatigue due to the exercise test order can explain the lack of difference between supplement and PL conditions in the majority of the variables. Previously performed exercises could reduce motor unit recruitment ability and increase metabolic ion (e.g., H^+^, ammonia) accumulation, especially in lower extremities [41]. On the other hand, it can be suggested that the current protocol mimics typical resistance training regimes, where limited amount of time is available between upper and lower body exercises. If that was true, a supplement could maintain higher muscle mean power output for longer periods of time. However, the efficacy of the supplement ingestion on short high-intensity exercise should be the subject of further studies. The authors did not examine the effect of every single ingredient alone, or the effect of different compositions of the substances used in the supplement; therefore, it cannot be stated which ingredient could be responsible for the potentially highest synergistic effect. Most studies that examined the various effects of taurine in combination with other ingredients did not use appropriate control supplement [38]. Therefore, taurine’s ability to enhance resistance exercise performance in human subjects remains unclear. Additionally, beta-alanine has been shown to significantly elevate carnosine levels in both type I and type II human muscle fibers and act as an intracellular buffer [15]. The recommended dose of beta-alanine loading is 2–5 g and a minimum 2–4 weeks of supplementation is needed to increase muscle carnosine levels [17]. However, it is currently still not known whether it is possible to enhance resistance exercise by acute beta-alanine ingestion.

Tyrosine supplementation is assumed to maintain optimum levels of brain neurotransmitters, which may contribute to the optimal performance through higher motivation levels together with decreased fatigue. However, in the study of Sutton et al. [18], even 30 times higher tyrosine dosage was insufficient to improve exercise performance. L-arginine and L-citrulline are believed to be potent precursors of nitric oxide (NO), which plays a crucial role in blood flow, muscle energy metabolism and mitochondrial oxidation during exercise [21,22]. In a review by Álvares et al. [43], only five acute studies evaluated L-arginine ingestion on exercise performance and only three of these reported a significant improvement. Dosage of 6 g of L-arginine 80 min before isokinetic elbow extension test did not reveal any significant changes [1]. Additionally, some studies have found that oral L-citrulline supplementation has no effect on exercise [42]. It must be noted that in light of the current evidence, a single dose of L-citrulline and L-arginine is insufficient to enhance sport performance, and supplementation should last at least one week [1,43]. Moreover, a review by Bescós et al. [44] indicates a paucity of data linking an increase in exercise performance and intake of NO–related supplements. Additionally, huperzine present in Huperzia serrata extract was reported to inhibit the acetylcholinesterase enzyme [25,26]. It should be noted that while manufacturers of dietary supplements are responsible for ensuring the safety of their products and accurate labeling that will not mislead the end consumer. At the same time, a manufacturer is not obligated to provide the Federal Drug Administration (FDA) or European Food Safety Agency (EFSA) with data demonstrating the safety and the effectiveness of the product before it is marketed [44,45,46]. Several studies have shown that pre-training supplements can potentially delay fatigue and improve the quality of resistance exercise [2,5]. However, in many of these studies a number of pharmacologically active compounds were mixed together, so it is impossible to assess the effectiveness of each component separately. In this case, the effectiveness of each single ingredient remains unclear [31]. This is because multiple ingredients potentially interact and these interactions may potentiate or attenuate supplement effectiveness. It is generally accepted that pre-workout supplement producers attempt to maximize the effectiveness of caffeine, while also offering several ingredients that attempt to further elevate its stimulatory potential.

Due to the lack of information in regards to the combination of the individual ingredients and their exact action in comparison with caffeine ingestion we are unable to identify the efficacy or whether those individual ingredients act synergistically or antagonistically with other compounds of MIPS. Further research will need to examine the effects of each individual ingredient of MIPS, and their combination with caffeine is needed to identify the most optimal composition regarding the choice of the appropriate active compounds and their dosage.

Despite the availability of a large amount of research also cited in this article pointing the improving endurance in resistance training, this study can be considered innovative as the former impact on endurance in resistance training has not been tested using the unique active ingredients composition of MIPS in connection with the protocols applied in the study, showing the holistic beneficial effect on the anaerobic capacity.

Limitation of the Study

In this study, we focused on the effect of a multi-ingredient supplement on anaerobic capacity. The base ingredient of the composition was caffeine. It is possible that similar effects could be observed for caffeine supplementation only or for another multi-ingredient composition similar to the one used in this study. However, the aim of this study was to determine whether the proposed combination and proportions of the ingredients in the supplement have a beneficial effect on the anaerobic capacity, and not to assess which component determines the effect to the greatest extent. The search for this ingredient or another combination or proportion of the components and evaluation of the optimal dose should be the subject of further research.

## 5. Conclusions

In conclusion, the results of this study indicate that the supplement KO significantly improves upper and lower body strength performance in resistance-trained men. At the same time, acute ingestion of this supplement had significant and beneficial effect on anaerobic power performance. Given the scarcity of research on pre-workout supplements, more research is warranted to gain a better understanding of their effects on anaerobic modes of exercise.

## Figures and Tables

**Table 1:** Mechanical variables obtained during isokinetic strength test at 60°/s for the right and left knee extensors and flexors. FL—flexion, EX—extension, TTP—time to peak torque, T@0.2 s—torque at 0.2 s, Twork—total work done.

Parameter		Right Leg			Left Leg		
	Movement	Placebo	Supplement	*p*	Placebo	Supplement	*p*
TTP [ms]	FL	548.7 ± 159.9	468.5 ± 141.3	0.002	615.2 ± 202.8	501.22 ± 170.1	0.002
	EX	501.7 ± 123.9	512.1 ± 110.2	0.818	539.6 ± 119.5	523.52 ± 123.4	0.422
T@0.2 s [Nm]	FL	103.2 ± 37.6	131.8 ± 29.3	0.001	103.7 ± 39.6	129.38 ± 28.4	0.001
	EX	202.6 ± 58.6	237. 2 ± 54.8	0.001	203.3 ± 63.2	229.84 ± 50.8	0.002
Twork [J]	FL	721.0 ± 150.2	798.1 ± 149.1	0.002	788.7 ± 145.1	843.18 ± 132.2	0.005
	EX	1172.4 ± 188.7	1337.0 ± 200.1	0.001	1327.2 ± 223.0	1419.52 ± 205.1	0.002

**Table 2:** Mean mechanical and physiological variables obtained during Wingate test. PP—peak power, MP—mean power, Twork—total work, FI—fatigue index. Significant difference compared to placebo was observed for MP (*p* = 0.038).

Parameter	Placebo	Supplement	*p*
PP [W/kg]	10.9 ± 0.8	11.1 ± 0.9	0.065
MP [W/kg]	8.5 ± 0.6	8.7 ± 0.5	0.038
Twork [kJ]	22.7 ± 2.7	23.1 ± 2.6	0.177
FI [%]	18.9 ± 4.0	19.4 ± 4.8	0.244
Lactate concentration [mmol/L]	14.6 ± 2.0	14.4 ± 1.7	0.873
